# Supplementary material for: Excessive premature mortality among children with cerebral palsy in rural Uganda: A longitudinal, population-based study
Source: PLoS One. 2020 Dec 29;15(12):e0243948. doi: 10.1371/journal.pone.0243948 (PMC7771855; doi:10.1371/journal.pone.0243948)
Supplement: S1 Table — NA = not available data; 3 children did not complete functional assessments, and one child had incomplete anthropometric measurements at first examination. Note that 6 children had chronic malaria infections as underlying COD. Sign “–”indicates no underlying COD. (DOCX) [file pone.0243948.s002.docx]

|  |  |  |  |  |  |  |
| --- | --- | --- | --- | --- | --- | --- |
|  |  |  |  |  |  |  |

| Age at 1^st^ exam (y) | Age at  death (y) | GMFCS  Level | Associated imp/seizure | Severe malnutrition | Immediate COD | Underlying COD |
| --- | --- | --- | --- | --- | --- | --- |
| 2·2 | 2·4 | NA | NA | 1 | Pneumonia | - |
| 2·3 | 2·4 | 5 | 1 | 0 | Anaemia | Malaria |
| 2·8 | 3·9 | 1 | 1 | 0 | Anaemia | Malaria |
| 3·4 | 5·8 | 5 | 0 | 1 | Malaria | - |
| 4·1 | 7·9 | 5 | 1 | 1 | Anaemia | Malaria |
| 4·8 | 4·9 | NA | NA | 0 | Malaria | - |
| 4·9 | 9·0 | 5 | 0 | 1 | Malaria | - |
| 5·8 | 9·7 | 5 | 1 | 1 | Anaemia | Malaria |
| 8·0 | 10·6 | 3 | 0 | 1 | Malaria | - |
| 9·8 | 12·9 | 3 | 1 | 1 | Pneumonia | - |
| 11·3 | 13·2 | 5 | 0 | 1 | Meningitis | - |
| 13·2 | 13·2 | NA | NA | 1 | Pneumonia | - |
| 14·0 | 17·4 | 1 | 1 | 0 | Meningitis | - |
| 16·9 | 18·8 | 2 | 1 | 1 | Anaemia | Malaria |
| 17·6 | 20·7 | 3 | 1 | NA | Anaemia | Malaria |

S1 Table

S1 Table legend:

Immediate COD and risk factors for the 15 deceased children with cerebral palsy. NA = not available data; 3 children did not complete functional assessments, and one child had incomplete anthropometric measurements at first examination. Note that 6 children had chronic malaria infections as underlying COD. Sign “–“ indicates no underlying COD.
